# *Aminobacter* MSH1-Mineralisation of BAM in Sand-Filters Depends on Biological Diversity

**DOI:** 10.1371/journal.pone.0128838

**Published:** 2015-06-15

**Authors:** Flemming Ekelund, Christoffer Bugge Harder, Berith Elkær Knudsen, Jens Aamand

**Affiliations:** 1 Dept. of Biology, Copenhagen University, Universitetsparken 15, DK-2100, Copenhagen Ø, Denmark; 2 Dept. Geochemistry, Geological Survey of Denmark & Greenland, Ø. Voldgade 10, DK-1350, Copenhagen, Denmark; Friedrich Schiller University, GERMANY

## Abstract

BAM (2,6-dichlorobenzamide) is a metabolite of the pesticide dichlobenil. Naturally occurring bacteria that can utilize BAM are rare. Often the compound cannot be degraded before it reaches the groundwater and therefore it poses a serious threat to drinking water supplies. The bacterial strain *Aminobacter* MSH1 is a BAM degrader and therefore a potential candidate to be amended to sand filters in waterworks to remediate BAM polluted drinking water. A common problem in bioremediation is that bacteria artificially introduced into new diverse environments often thrive poorly, which is even more unfortunate because biologically diverse environments may ensure a more complete decomposition. To test the bioaugmentative potential of MSH1, we used a serial dilution approach to construct microcosms with different biological diversity. Subsequently, we amended *Aminobacter* MSH1 to the microcosms in two final concentrations; i.e. 10^5^ cells mL^-1^ and 10^7^ cells mL^-1^. We anticipated that BAM degradation would be most efficient at “intermediate diversities” as low diversity would counteract decomposition because of incomplete decomposition of metabolites and high diversity would be detrimental because of eradication of *Aminobacter* MSH1. This hypothesis was only confirmed when *Aminobacter* MSH1 was amended in concentrations of 10^5^ cells mL^-1^.Our findings suggest that *Aminobacter* MSH1 is a very promising bioremediator at several diversity levels.

## Introduction

BAM (2,6-dichlorobenzamide), a metabolite of the pesticide dichlobenil and a common groundwater contaminant, is considered a serious problem in drinking water supply [1]. Very few bacteria can degrade BAM, and BAM degraders are not common in natural systems [2,3]. *Aminobacter* MSH1 is a candidate for a bacterium, which can be amended to sand filters in waterworks to remediate BAM-polluted drinking-water [4,5]. However, it is not straightforward to amend non-indigenous bacteria to already existing microbial communities. For decades, we have known that bacteria artificially introduced into new ecosystems often rapidly decline in numbers [6]. This fact, which is a major obstacle to the use of non-indigenous bacteria for remediation purposes, has been attributed to various factors which include the scarcity of available nutrient sources and the hostility of the non-native environment due to many different biotic and abiotic factors [7]. Cutler [8] already in 1923 demonstrated that protozoan predation bears a great responsibility for the disappearance of artificially amended bacteria, which has subsequently repeatedly been demonstrated experimentally; e.g. [9]. Competition from other bacteria also plays an important role [7], and probably also predatory bacteria and bacteriophages [10]. These factors, however, are not experimentally investigated to the same extent.

Conversely, a complex community may also facilitate degradation of organic pollutants. Degradation processes are often performed in collaboration; i.e. by consortia of microorganisms. Often microbial consortia degrade recalcitrant compounds more efficiently than pure cultures because their biodiversity can increase the number of catabolic pathways available for the biodegradation of the pollutant in focus. A well-functioning consortium is also likely to survive better when transferred to a new environment than a single strain [11–13]. In particular, the presence of protozoan grazers will stimulate bacterial degrading activities, if the grazing pressure is not too severe [6]. Mattison et al. [14], for example, reported that a *Pseudomonas* strain degraded benzene aerobically at a rate three times higher in the presence of the flagellated protozoan *Heteromita globosa* than in its absence; the rate was even higher when the protozoa grew exponentially. Similarly, Tso and Taghon [15] found that the indigenous microbial community in contaminated marine sediment mineralized significantly less naphthalene after addition of Cytochalasin B, a compound which inhibits protozoan but not bacterial activity.

Despite new state of the art sequencing methods, an exhaustive description of the full diversity even in a single gram of soil is still not possible because it contains several thousands of different taxonomic taxa [16] most of which have never been described. Moreover, literally millions of different possible interactions can take place between these taxa. Therefore, we choose to approach soil diversity merely statistically. The thousands of different taxonomic taxa in a gram of soil will be distributed over billions of individuals and the relationship between number of individuals and number of taxa in a particular smaller subsample can be described by a rarefaction curve [17]. Thus, a series of subsamples containing increasingly fewer individuals will also contain increasingly fewer taxa. An indication of how number of taxa will decrease with increasing serial dilution can be obtained from the logarithmic relationship S = α*ln(N/α), where S is species number, N the number of individuals and α is a measure of diversity [18]. Thus when we dilute serially, the number of taxa will roughly decrease in a linear fashion.

Here, we report an experiment where we studied the ability of *Aminobacter* MSH1 to establish, persist and hence degrade BAM in sand-microcosms with different initial biological diversity. We established a 12 step consecutive diversity gradient from soil, running from almost “full soil diversity” to systems with very low diversity. We produced the diversity gradient by serial dilution of unsterile pasture soil and let the dilutions grow for a period with a growth medium as to facilitate difference only in diversity and not in biomass. We then amended the sand-microcosms with MSH1 in concentrations of either 10^5^ or 10^7^ cells mL^-1^. Concomitantly we added a bacterial growth medium to the microcosms as in [19] who showed that both diversity reduction and nutrient addition would facilitate establishment of a non-indigenous bacterium. We supplied BAM to the systems twice, first concomitantly with the addition of the bacteria and again after four weeks to examine persistence of MSH1.

Our purpose was to identify the diversity level at which the conditions for survival and activity of MSH1 were optimal. Furthermore, we wanted to investigate whether MSH1 was able to actively degrade BAM in a complex community. We stress that here we understand the concepts “diversity” and “optimal conditions” as statistical quantities. Specifically, we hypothesized that: 1) BAM-degradation would be most efficient in systems with an “intermediate diversity”, because MSH1 would have difficulty establishing itself at high diversities due to predation/competition-pressure; whereas at low diversities, degradation would be less efficient due to low metabolic diversity. 2) Secondly, we expected that in systems inoculated with 10^7^ mL^-1^, *Aminobacter* MSH1, BAM would be degraded more efficiently. 3) Thirdly, for statistical reasons we expected persistence of MSH1 to be higher when 10^7^ mL^-1^ bacteria were added due to the increasing likelihood that some of the bacterial cells avoided competition or predation, and thus established.

## Materials and Methods

Below we give an account of how the experiment was performed. To increase clarity, we have included Table 1, which briefly summarizes the experiment in a schematic form.

**Table 1:** Schematic outline of the experiment.

Experimental day	Day 1	Day 1–21	Day 21	Day 21–49	Day 49	Day 49–77
Microcosm age			Day 1	Day 1–28	Day 28	Day 28–56
	Preparation of soil dilutions	Growth of micro-bial cultures in TSB	Start of sand-micro-cosms: 1^st^ spiking with BAM	1^st^ round with sand-microcosm with different diversity	2^nd^ additional spiking with BAM	2^nd^ experi-mental round with sand-microcosms
	Start of serially diluted cultures in TSB	Regular measure-ment of ^14^CO_2_ to estimate activity in cultures	Sampling of TSB-cultures for bac-teria and protozoa	Regular measurement of ^14^CO_2_		Regular measurement of ^14^CO_2_

The whole experiment lasted 77 days. We used the first 21 days to produce cultures with different diversity but comparable biomass. At experimental day 21, we started the sand-microcosms, containing a mixed microbial communities, ^14^C-labelled BAM, and the BAM-degrader MSH1. During the 1^st^ experimental round (Experimental day 21–49, Microcosm day 1–28) we regularly measured ^14^CO_2_ to estimate BAM-degradation. At experimental day 49 (Microcosm day 28), we spiked the microcosms with BAM once more. During the 2^nd^ experimental round (Experimental day 49–77, Microcosm day 28–56) we continued regular measurement of ^14^CO_2_.

### Preparation of soil inocula with different initial biological diversities

To prepare inocula with different initial microbial community-diversities, we mixed 10 g unsterile pasture soil from Löddeköpinge, Sweden [19] with 10 ml 0.1 g L^-1^ sterile dilute Tryptic Soy Broth. From this suspension (dilution 1), we made repeated successive transfers of 5 ml soil suspension to 20 ml dilute Tryptic Soy Broth in sterile 116 ml serum-flasks. Thus, we prepared 14 successive, 5-folds dilutions of the soil slurry. The amount of soil in the dilutions ranged from 5x10^2^ g L^-1^ in the lowest dilution (dilution 1) to 4.1x10^-7^ in the highest dilution (dilution 14). After preparation, we closed the flasks with air-tight rubber-stoppers and sealed them. We incubated the sealed flasks in the dark at 15°C and measured CO_2_- accumulation regularly by injecting 1 ml headspace-air into a gas chromatograph with a thermal conductivity detector (Mikrolab Aarhus) to get an indication of community activity. After three weeks, when the CO_2_-production had levelled out, we considered the inocula to be ready. The rationale behind this procedure is that it will produce communities with very different biological diversity but similar, if not completely identical, microbial biomass [20].

### Bacterial and protozoan measurements

At the end of the incubation period we measured bacterial CFU’s on Tryptic Soy Agar (Bacto, Becton, Dickinson and Company, New Jersey, USA) in the 14 dilutions as in [21]. Protozoa were enumerated as in [22]: In short, we used a most probable number (MPN) approach. A soil suspension, 5.0 g of soil in 100 ml phosphate buffer, was successively 3-fold diluted. We inoculated the successive dilutions in 8x12 well microtitre plates (Sarstedt, Newton, USA) with Tryptic Soy Broth medium (0.1 gL^−1^). The plates were stored in darkness at 15°C and the wells were examined after one and three weeks using an inverted microscope.

### Construction of sand microcosms

After the three-week stabilization period, we prepared six microcosms from each of the 12 lowest dilutions; the two highest dilutions (13 and 14) contained no bacteria and were not used further. Initially we constructed each microcosm from 10 g of sterile quartz-sand and 2 ml suspension from one of the 12 microbial dilutions. We then amended three of the microcosms from each dilution with 0.25 ml of a 10^6^ cells mL^-1^ suspension of the BAM-degrader MSH1 in an MSNC medium [23], whereas the other half were amended with 100 times as many bacteria, i.e. 0.25 ml of a 10^8^ cells mL^-1^ suspension. Finally, we amended each microcosm with 0.25 ml dilute Tryptic Soy Broth (0.1 g L^-1^) containing 0.1 mg L^-1^ [*ring*-U-^14^C] BAM (25.2 mCi mmol^-1^) purchased from International Izotop (Budapest, Hungary). After setup, the microcosms contained 10 μg L^-1^ BAM; half of the microcosms contained 10^5^ MSH1-cells mL^-1^ and the other half 10^7^ MSH1-cells mL^-1^. We further prepared three microcosms from the strongest soil dilution (dilution 1) as described above but with buffer devoid of MSH1-cells; this was to document that no indigenous bacteria actually degraded BAM. In summary, we prepared 12 microcosms in triplicates for two MSH1-concentrations and three control-microcosms without MSH1; i.e. a total of 75 microcosms.

### Measurement of ^14^CO_2_ from BAM-degradation

We supplied each microcosm with a 10-ml glass tube containing 1.0 ml 1 M NaOH to trap ^14^CO_2_ evolved from the BAM-mineralisation. The NaOH was replaced on day 1, 2, 4, 7, 15, 21 and 28 after microcosm setup, mixed with 10 ml Wallac OptiPhase HiSafe 3 scintillation cocktail (Turku, Finland), and counted for 10 min in a Wallac 1409 liquid scintillation counter [24]. At day 28 after experimental start, we spiked the microcosms again with 0.1 mg L^-1^ BAM dissolved in 0.25ml Tryptic Soy Broth (0.1 g L-1) and the experiment was continued for another 28 days.

## Results and Discussion

**Fig 1:**
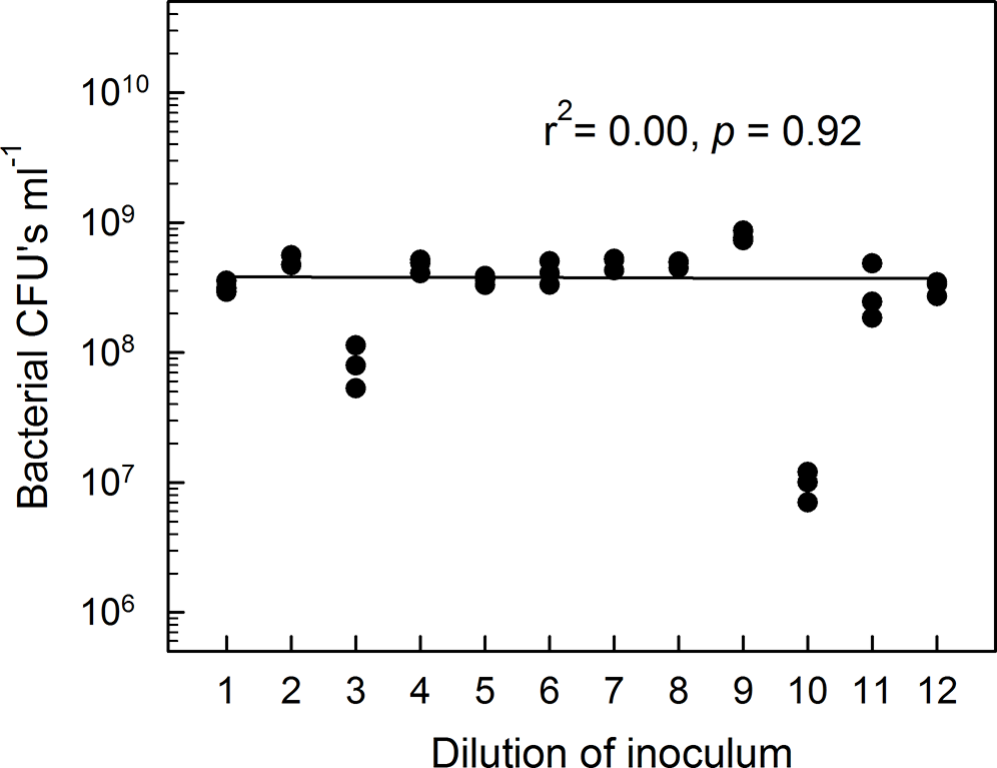
The number of bacterial colony forming units (CFU’s) recorded from 116 ml serum-flasks containing 20 ml sterile Tryptic Soy Broth 21 days after the flasks were inoculated with soil suspensions. These flasks were used to inoculate our microcosms. The soil suspensions were successively 5-fold diluted. We found no correlation between dilution level of inoculum and CFU-number after 21 days (r^2^ = 0.00, *p* = 0.92).

By a simple dilution process we examined the effect of biological diversity in complex communities on the ability of *Aminobacter* MSH1 to degrade BAM. The rationale behind this approach is that microcosms inoculated with increasingly more dilute soil-suspensions will contain increasingly less microbial diversity. Franklin et al. [25] and Wertz et al. [26] used similar dilution approaches to demonstrate that colony morphology and DGGE-based taxon-richness decreased with dilution. Likewise, Yang et al. [27] showed that the functional diversity as indicated by BIOLOG ECO-plate analysis declined significantly with dilution. The procedure, we used for preparation of inocula of different taxon richness, was intended to produce inocula that contained approximately the same bacterial biomass irrespective of taxon richness [20]. We reckon that we succeeded reasonably well in accomplishing this goal. We recorded on average 3.8×10^8^ CFU’s mL^-1^ in the inoculum suspensions and found no indication that bacterial density in the soil suspensions used for inoculation of the microcosms correlated at all (positively or negatively) with the original dilution level (r^2^ = 0.00; P = 0.92; Fig 1). In dilutions 3 and 10, though, the number of CFU’s deviated significantly from this average value (Fig 1). Likely explanations are that the communities here were dominated by large morpho-types, hence their biomass was contained in fewer cells, or that these dilutions contained a higher fraction of forms, which could not be cultured on the offered medium.

**Fig 2:**
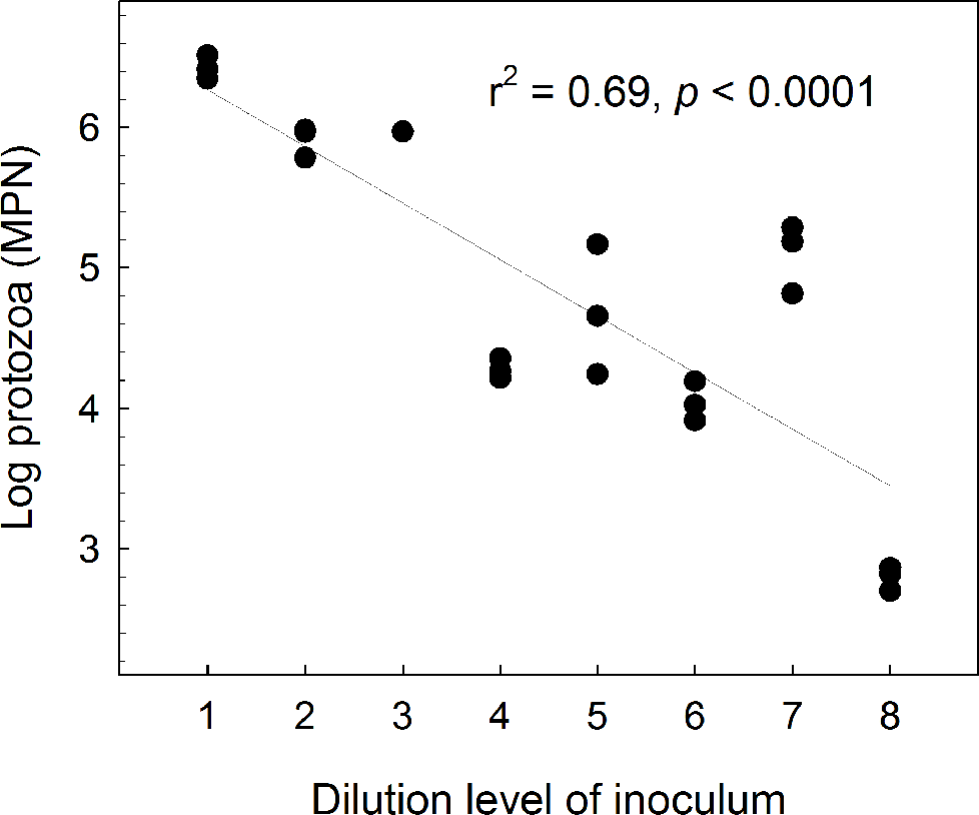
The most probable number of protozoa (MPN) in 116 ml serum-flasks containing 20 ml sterile Tryptic Soy Broth amended with successively 5-fold diluted soil suspension. Flasks were incubated for 21 days. We found no protozoa in flasks above dilution level 8. From dilution level 1 to 8, there was a negative correlation between dilution level of inoculum and MPN after 21 days (r^2^ = 0.69, *p* < 0.0001). We only obtained one data-point for dilution 3 as two samples were lost.

We also measured protozoan density in the original inocula-flasks. By contrast to the bacteria, protozoan numbers did actually decrease with the dilution. We only recovered protozoa from dilutions 1–8. Here, protozoan numbers correlated negatively with dilution level (r^2^ = 0.69; P <0.0001; Fig 2). Usually soil contains less than 10^5^ protozoan cells per gram [6], hence the complete lack of protozoa in the four highest dilutions was expected, which further stresses that the dilution approach worked. Protozoa are highly selective in their choice of food [20], and as the number of bacterial taxa decrease with dilution, it is likely that certain bacteria with a particularly high food-quality were absent from the highest dilutions. This may explain the significant decrease in protozoan numbers with dilution. This is accordance with [28], who found that growth rate of the nematode *Caenorhabditis elegans* increased with prey richness. This was primarily because the best available prey species was absent in less diverse consortia, thus growth of *C*. *elegans* feeding on a prey-mixture was approximately equal to growth on the single best prey in monoculture. This presumed depletion of food-quality with dilution is further supported by the very low protozoan number in dilution 8 (Fig 2). We emphasize that the low bacterial numbers in dilution 10 cannot be ascribed to protozoan grazing, as no protozoa were found in this dilution (Figs 1 and 2).

**Fig 3:**
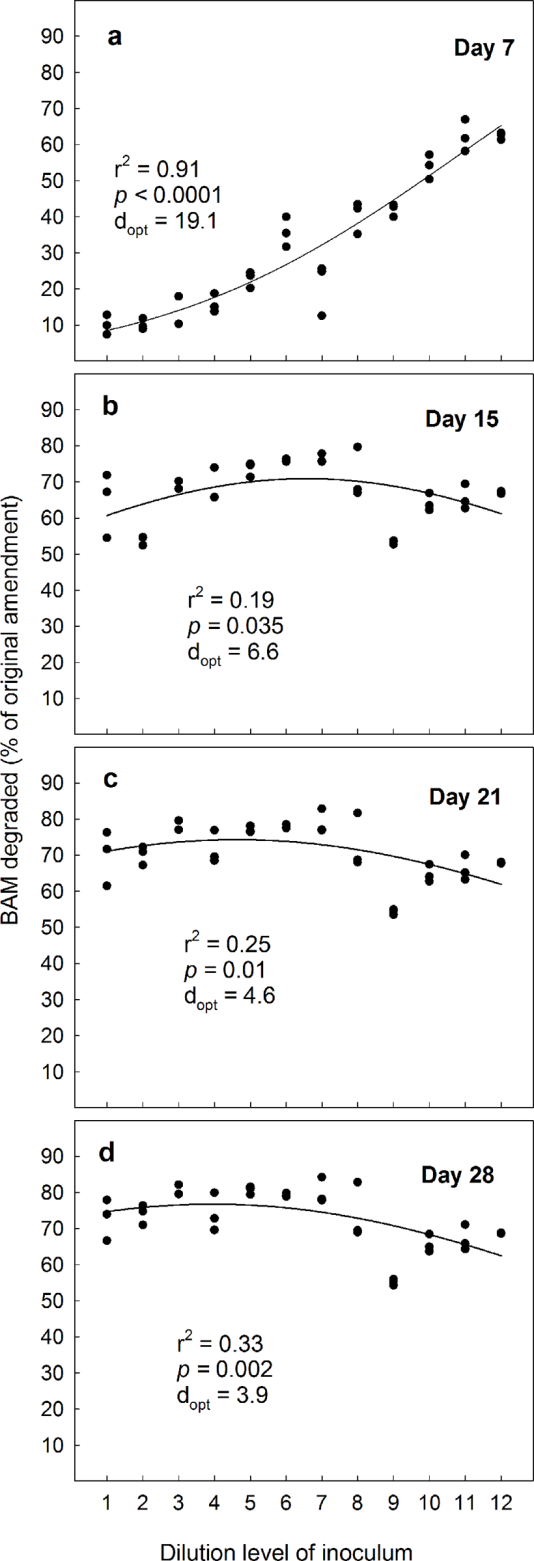
The ability of the bacterium Aminobacter MSH1 to establish in sand microcosms and degrade the pesticide-residue BAM when inoculated in concentrations of 10^5^ cells L^-1^. Degradation-efficiency was measured as cumulated ^14^CO_2_ in % of original amendment (10 μg L^-1^ BAM) on four sampling-days during a 28 day experimental period. Microcosms were inoculated with a gradient of decreasing biological diversity, obtained by serial dilution and regrowth of soil to facilitate a similar biomass irrespective of diversity. The dilution series covered a span of diversity from “almost full soil diversity” to nearly 0. We fitted data to a simple bell-shaped Gaussian curve; y = a*exp(0.5*((x- d_opt_)/b)^2^), to test the underlying hypothesis that BAM-degradation would be most efficient at intermediate diversity-levels. The value of “d_opt_”is the dilution level where the equation suggests that BAM-degradation is most efficient. In the equation, the value of “a” represents a hypothetical maximum degradation-percentage, whereas “b” is a measure of how the degradation values vary around “d_opt_“.

*Aminobacter* MSH1 is one of the few microorganisms that can degrade BAM [24] and, as we expected, microcosms without MSH1-amendment displayed no degradation at all (data not shown). Conversely, all systems amended with MSH1 had a vigorous BAM-degradation. At day 7, microcosms amended with 10^5^ MSH1 mL^-1^ displayed the highest BAM-degradation in systems with the lowest initial diversity (Fig 3A). Thus, MSH1 amended at this low density established itself best initially where the environment contained the lowest diversity (Fig 3A). There has been much focus on the importance of protozoan grazing for disappearance of inoculated non-indigenous bacteria [6,9]. Less attention has been paid to competition from other bacteria, which probably also plays an important role [7]. Even an environment as simple as a static liquid culture provides multiple ecological niches, where fierce competition between bacteria takes place [29]. Attack from predatory bacteria (*Bdellovibrio* and similar organisms) and bacteriophages may also reduce bacterial populations [10]. The quantitative ecological role of these organism-groups in soil-systems is, however, poorly understood.

Our fundamental hypothesis was that BAM-degradation would be most efficient in systems with “intermediate diversity” because two different oppositely working factors would impede the degradation. At higher diversities, we expected numbers of MSH1 to be reduced due to numerous interactions with indigenous micro-biota through predation, competition etc. At lower diversities, the number of catabolic pathways (Siripattanakul et al. 2009) would be reduced and the protozoan stimulation [14] would not be an activity-enhancing factor. To test this, we consequently fitted our data to a simple bell-shaped Gaussian curve; y = a*exp(0.5*((x- D_opt_)/b)^2^). This is an exact way to test the hypothesis and it will provide an evidence-based, numerical estimate of the dilution/diversity level where the most efficient degradation takes place (D_opt_). In the first experimental round with MSH1 added in a concentration of 10^5^ cells mL^-1^, we observed that during the 28 day experimental period D_opt_ moved from right to left along the x-axis; i.e. from a higher dilution-level (and thus a lower diversity-level) to a lower dilution-level (and thus a higher diversity-level) (Fig 3A–3D). This suggests that the later stages of the degradation were best performed in systems where MSH1 was accompanied by a larger suite of organisms.

The accumulation of ^14^CO_2_ from mineralisation of BAM by *Aminobacter* MSH1 normally reaches a plateau at levels about 15–64% of the initial added ^14^CO_2_-radio labelling in pure culture depending on the initial BAM concentration and culture conditions [24]. Remaining ^14^C in the liquid medium may then be present as ^14^C-labelled degradation products, cell exudates or incorporated into the MSH1 biomass [24]. We suggest that further mineralisation of these radiolabelled products and also new exudates produced during decay of starving MSH1 cells are facilitated better by diverse microbial communities.

**Fig 4:**
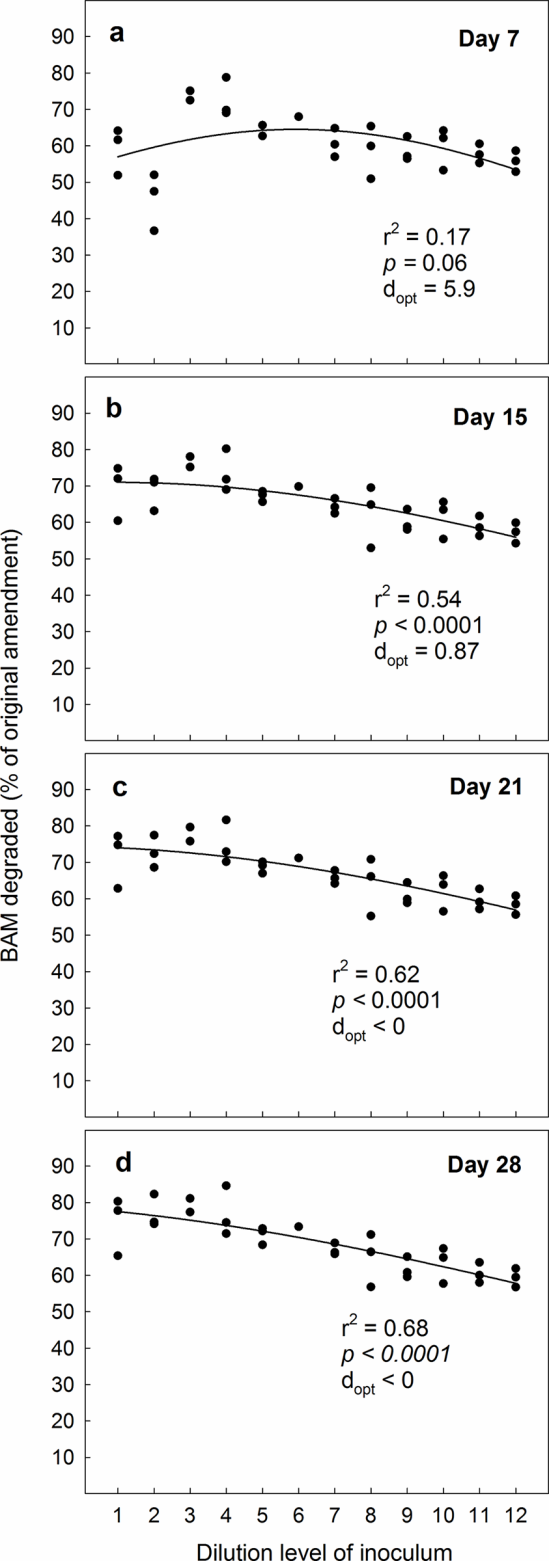
The ability of the bacterium Aminobacter MSH1 to establish in sand microcosms and degrade the pesticide-residue BAM when inoculated in concentrations of 10^7^ cells L^-1^. Degradation-efficiency was measured as cumulated ^14^CO_2_ in % of original amendment (10 μg L^-1^ BAM) on four sampling-days during a 28 day experimental period. Microcosms were inoculated with a gradient of decreasing biological diversity, obtained by serial dilution and regrowth of soil to facilitate a similar biomass irrespective of diversity. The dilution series covered a span of diversity from “almost full soil diversity” to nearly 0. We fitted data to a simple bell-shaped Gaussian curve; y = a*exp(0.5*((x- d_opt_)/b)^2^), to test the underlying hypothesis that BAM-degradation would be most efficient at intermediate diversity-levels. The value of “d_opt_”is the dilution level where the equation suggests that BAM-degradation is most efficient. In the equation, the value of “a” represents a hypothetical maximum degradation-percentage, whereas “b” is a measure of how the degradation values vary around “d_opt_“.

In line with our second hypothesis, we found that when the microcosms spiked with BAM were amended with 10^7^ MSH1-cells mL^-1^ optimal degradation took place already initially at a higher diversity level than when microcosms were amended with 10^5^ MSH1-cells mL^-1^ (Fig 4A). Likewise, D_opt_ moved further to the left (Fig 4A–4D) during the experiment. At day 28, the differences in degradation efficiency between microcosms amended with high and low densities of MSH1 levelled out, although D_opt_ consistently was lower in systems with 10^7^ MSH1-cells mL^-1^; compare Figs 3D and 4D. Still, averagely taken over all dilutions, significantly more BAM was degraded at the 10^5^ cells mL^-1^ amendment than at the 10^7^ cells mL^-1^ amendment; i.e. 72.4% vs. 68.5% (p = 0.04, One-way Anova), which contradicts our second hypothesis that BAM would be degraded more effectively at high MSH1 densities. Possibly, as degradation takes place most efficiently through the collaboration between different organisms, the systems amended with 10^7^ cells mL^-1^ MSH1 became dominated by MSH1 to the extent that it might have hampered other microorganisms. We note though, that while the systems amended with 10^5^ cells mL^-1^ showed gradually increasing mineralisation with increasing dilution on day 7 from c. 10% in the most diverse to >60% in the least diverse microcosms (Fig 3A), the systems amended with 10^7^ cells mL^-1^ had reached BAM mineralisation levels of >50% in all dilutions on day 7 (Fig 4A). This suggests that if MSH1 is to be used in systems with continuous culture (such as e.g. waterworks sand filters), where the microorganisms are washed out continuously, high densities of MSH1 are still preferable to lower densities; in such environments. The most important factor of a bioremediating bacterium is not the total accumulated mineralisation effect over weeks, but rather its ability to grow up quickly and establish itself.

**Fig 5:**
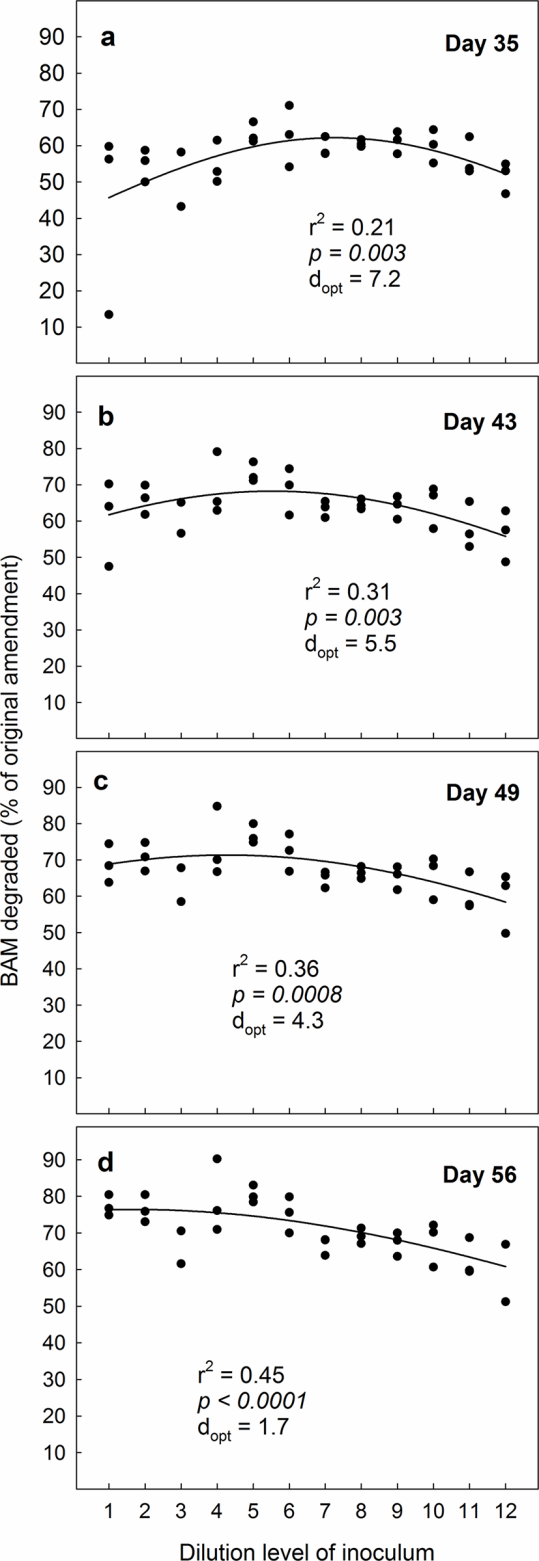
The ability of the bacterium Aminobacter MSH1 to persist in sand microcosms and degrade the pesticide-residue BAM when originally inoculated in concentrations of 10^5^ cells L^-1^. Microcosms were first spiked with BAM and followed for a 28 day period (see Fig 3); subsequently they were spiked again and followed for another 28-day period. Degradation-efficiency was measured as cumulated ^14^CO_2_ in % of original amendment (10 μg L^-1^ BAM) on four sampling-days. Microcosms were originally inoculated with a gradient of decreasing biological diversity, obtained by serial dilution and regrowth of soil to facilitate a similar biomass irrespective of diversity. The dilution series covered a span of diversity from “almost full soil diversity” to nearly 0. We fitted data to a simple bell-shaped Gaussian curve; y = a*exp(0.5*((x- d_opt_)/b)^2^), to test the underlying hypothesis that BAM-degradation would be most efficient at intermediate diversity-levels. The value of “d_opt_”is the dilution level where the equation suggests that BAM-degradation is most efficient. In the equation, the value of “a” represents a hypothetical maximum degradation-percentage, whereas “b” is a measure of how the degradation values vary around “d_opt_“.

**Fig 6:**
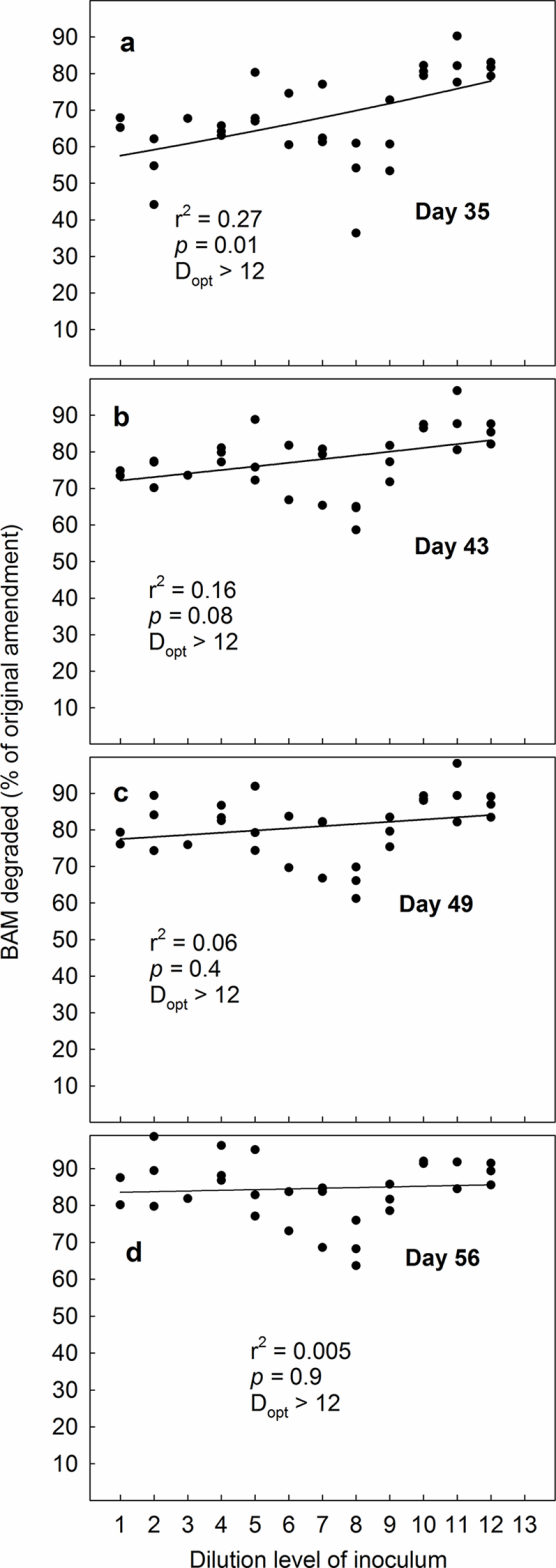
The ability of the bacterium Aminobacter MSH1 to persist in sand microcosms and degrade the pesticide-residue BAM when originally inoculated in concentrations of 10^7^ cells L^-1^. Microcosms were first spiked with BAM and followed for a 28 day period (see Fig 3); subsequently they were spiked again and followed for another 28-day period. Degradation-efficiency was measured as cumulated ^14^CO_2_ in % of original amendment (10 μg L^-1^ BAM) on four sampling-days. Microcosms were originally inoculated with a gradient of decreasing biological diversity, obtained by serial dilution and regrowth of soil to facilitate a similar biomass irrespective of diversity. The dilution series covered a span of diversity from “almost full soil diversity” to nearly 0. We fitted data to a simple bell-shaped Gaussian curve; y = a*exp(0.5*((x- d_opt_)/b)^2^), to test the underlying hypothesis that BAM-degradation would be most efficient at intermediate diversity-levels. The value of “d_opt_”is the dilution level where the equation suggests that BAM-degradation is most efficient. In the equation, the value of “a” represents a hypothetical maximum degradation-percentage, whereas “b” is a measure of how the degradation values vary around “d_opt_“.

To examine the persistence of MSH1 in the microcosms, we conducted an additional spiking of the microcosms with BAM after 28 days. When the microcosms originally amended with 10^5^ MSH1-cells mL^-1^ received an additional dose of BAM, we observed that optimal conditions for degradation were at a higher diversity level (i.e. a lower dilution level) than when we originally started the experiment (Figs 3A vs. Fig 5A). We attribute this to establishment and growth of MSH1 on BAM or components in the Tryptic Soy Broth during the preceding 28 day period. After the 28-day second experimental period, the optimum diversity level was likewise higher than in the first round (Fig 5A–5D). Surprisingly, when we spiked the microcosms originally amended with 10^7^ MSH1-cells mL^-1^ with an additional dose of BAM, we observed that the strongest BAM-degradation took place at the lowest diversity level; i.e. at the highest dilution level (Fig 6A). This prevailed throughout the experiment, albeit not at a significant level at day 21 and 28 (Fig 6A–6D). While we cannot provide any certain explanation for this phenomenon, we suggest that the amendment with the high concentration of MSH1 caused a negative feedback where growth of protozoa, predatory bacteria and bacteriophages was facilitated. These organisms were then responsible for reduction in numbers and activity of MSH1. Since the initial populations of predators on MSH1 declined with increasing dilution this negative feedback would likewise decrease. Nevertheless, as opposed to the first round, taken over all concentrations, significantly more BAM was degraded at the higher 10^7^ cells mL^-1^ amendment than at the 10^5^ cells mL^-1^ amendment; i.e. 84.5% vs. 70.9% (p <0.001, One-way Anova), which supports our third hypothesis that the persistence of MSH1 was higher at higher inoculum density.

We stress that we did not expect biological diversity as such to be the only factor that determined BAM-degradation. Specifically, particular factors beyond our control might also influence the process, such as random dominance or absence of certain organisms in some of the dilutions. This is exemplified by the low bacterial numbers in dilutions 3 and 10 (Fig 1). We have only been able to demonstrate some overall patterns in our results because we included so relatively many different diversity levels in our experiments. Some of our particular dilutions did not fit into the general picture; e.g. dilution 9 in Fig 3. Hence, we stress that the picture that we present is stochastic and not causal.

## Conclusions

We have demonstrated that MSH1 is able to colonize, establish and even persist in a complex community, which we attribute to its unique ability to degrade BAM. When MSH1 was amended in the low concentration of 10^5^ cells mL^-1^, the best degradation results were obtained at some intermediate diversity-level between the two extremes, i.e. nearly full soil diversity, and a system virtually without microorganisms. When MSH1 was amended in the high concentration of 10^7^ cells mL^-1^, the outcome was more ambiguous. Our findings should be encouraging for the ambitions to use MSH1 for bioaugmentative purposes, especially on waterworks sand filters with a microbial diversity that is lower than in soil but still higher than in our highest dilutions [30].

## Supporting Information

S1 Data (XLSX)

S2 Data (XLSX)

S3 Data (XLSX)

S4 Data (XLSX)

S5 Data (XLSX)

S6 Data (XLSX)
